# Single-Port Retroperitoneal Pancreatic Necrosectomy for the Treatment of Extrapancreatic Walled-Off Necrotic Collections

**DOI:** 10.1097/AS9.0000000000000019

**Published:** 2021-01-19

**Authors:** Rebecca Saunders, John P. Neoptolemos, Faye Hughes, Paula Ghaneh, Christopher M. Halloran

**Affiliations:** From the * Department of Molecular & Clinical Cancer Medicine, University of Liverpool, UK; † Department of General Surgery, University of Heidelberg, Germany; ‡ Department of General and Pancreatic Surgery, Liverpool University Hospitals NHS Foundation Trust, UK.

## Abstract

Mini-abstract. Single-port retroperitoneal pancreatic necrosectomy (SPRPN), a novel method to debride extrapancreatic necrosis after failed conventional treatment, was undertaken in 7 patients with a median collection diameter of 98 × 85 × 124 mm^3^, with resolution at a median of 42 days and postoperative median stay of 47 days.

Supplemental Digital Content is available in the text.

## INTRODUCTION

Endoscopic or minimally invasive step-up approaches are considered the gold standard of treatment for pancreatic collections or necrosis,^1,2^ with multiple techniques described.^3–6^ Unfortunately, extrapancreatic necrosis, which is separate and remote from the pancreas, is often not adequately accessible by standard methods, typically when either in or extending down the paracolic gutters.^7,8^ Overall, the management of extrapancreatic necrosis is not well addressed in the literature. Traditionally, treatment is by percutaneous drainage using relatively small diameter pigtail catheters inserted under radiologic guidance. Results are often unsatisfactory meaning open necrosectomy is required to debride the area, which in turn requires large incisions and sometimes multiple reoperations.

We have developed single-port retroperitoneal pancreatic necrosectomy (SPRPN), a novel method for managing extrapancreatic necrosis. A SILS port is placed into the necrotic collection and an articulating grasper (SILS Clinch) is used for debridement of necrotic tissue, providing a 300^o^ operational arc around the port. This study reports our initial experience with this technique in 7 consecutive patients.

## METHODS

### Patient Population

Definitions used were that of the Atlanta criteria.^9^ Acute pancreatitis was managed according to International Association of Pancreatology/American Association of Pancreatology guidelines.^10^ All patients were discussed by the multidisciplinary team, attended by pancreatic surgeons, gastroenterologists, endoscopists, and interventional radiologists. Intervention was reserved for patients with documented or suspicion of infected pancreatic necrosis or those with persistent pain or symptoms after an extended period of hospitalization. Infected necrosis was defined as positive tissue cultures or gas in the collection on computerized tomography (CT) imaging. Repeat CT was performed if there was clinical deterioration, a significant rise in inflammatory markers or for planning possible intervention. SPRPN was used in patients with symptomatic extra-pancreatic walled-off necrosis (WON) in either the flank or the paracolic gutters, or complex collections that would otherwise require an open necrosectomy. Patients were followed up regularly in outpatient clinic following discharge.

### Technique

Percutaneous drains (PCDs) were radiologically inserted into the necrotic collection. SPRPN was performed under general anesthetic. Ureteric stents were inserted prior to necrosectomy to protect against ureteric injury and to prevent traction of the collapsing cavity from kinking the ureters. The 3-channel SILS port (Medtronic/Covidien, CT) was inserted using an open cut down technique, where a 3-cm incision was centered over the PCD insertion point. A zero-degree nephroscope was inserted through one port and an articulating grasper (SILS-Clinch) through another. Continuous 0.9% saline irrigation was used through the nephroscope to expand the cavity. Near complete necrosectomy, so as to avoid hemorrhage, was undertaken by direct vision. Tissue samples were sent to microbiology for culture and sensitivities. A 10 or 12 Fr nasogastric tube was sutured to a corrugated drain and inserted into the cavity allowing postoperative irrigation and drainage, initially at a rate of 125 ml/h and reduced according to clinical response. Patients underwent further CT scans or necrosectomy procedures if clinically indicated by ongoing sepsis or nonresolution of the collection.

## RESULTS

Between December 2016 and September 2019, 7 patients with a median (interquartile range [IQR]) age of 59 (40–68) years underwent SPRPN (Table 1). Five patients were transferred from district hospitals to our supraregional center. The etiology was gallstones in 2 patients, ethanol in 2 patients and endoscopic retrograde cholangiopancreatography (ERCP) related in 3 patients of which 2 also had perforation of the second part of the duodenum. All patients had severe pancreatitis, all had infected necrosis, and 2 patients had multiorgan dysfunction syndrome. All patients had WON, 4 patients had predominantly right-sided collections, 2 predominantly left-sided collections, and 1 with a complex horseshoe collection involving both paracolic gutters. The median (IQR) dimensions of the collections (width × depth × height) were 98 mm (92–128) × 85 mm (72–110) × 124 mm (112–300). The first patient underwent 2 procedures as part of the learning curve, while the remaining patients underwent only 1 procedure each. The fourth patient had an infarcted right colon secondary to the inflammatory occlusion of mesenteric vessels, which was noted after the initial necrosis was cleared and was converted to an open necrosectomy with extended right hemicolectomy. Two patients required further PCD on subsequent admissions for further attacks of pancreatitis, unrelated to the original collections.

**Table 1.** Clinical Characteristics of Patients

		Patients							Summary Statistic	
	Patient Number	1	2	3	4	5	6	7	Median	IQR
Demographics	Age (y)	24	59	63	40	71	68	58	59	40–68
	Sex	F	F	F	M	M	M	F	—	—
	Etiology	Post-ERCP*	Post-ERCP*	Gall Stones	Ethanol	Post-ERCP	Gall stones†	Ethanol	—	—
	Smoker	N	Y	N	N	N	N	N	—	—
	Transfer to regional center	Y	Y	N	Y	Y	N	Y‡	—	—
	MODS	Y	N	N	Y	N	N	N	—	—
	Infected necrosis	Y	Y	Y	Y	Y	Y	Y	—	—
	Severity of pancreatitis	Severe	Severe	Severe	Severe	Severe	Severe	Severe	—	—
	Comorbidity	None	None	None	None	None	Cold agglutin disease	None	—	—
									Median	IQR
Imaging findings	Focus of WON	Right Paracolic	Right Paracolic	Right Paracolic	Right Paracolic and Right Upper Quadrant	Left Paracolic and Left Groin	Horseshoe	Left Paracolic		
	Pancreatic necrosis	N	N	N	N	Pancreatic tail (30%)	N	N	-	-
	Width (mm)	113	98	92	128	92	298	70	98	92–128
	Depth (mm)	110	85	59	73	97	207	72	85	72–110
	Height (mm)	124	142	63	112	300	316	113	124	112–300
									Median	IQR
Outcomes	Overall length of Stay (d)	61	133	88	98	108	217	19	98	61–133
	Time to PCD (d)	23	29	62	34	19	23	7	23	19–34
	Time from PCD to SPRPN (d)	12	2	7	17	30	73	2	12	2–30
	Number of sessions	2	1	1	1	1	1	1	—	—
	Postoperative length of stay (d)	23	102	19	47	59	120	13	47	19–102
	Postoperative time to resolution (d)	37	42	36	83	49	45	11	42	36–49
	Follow-up (mo)	39	6	30	24	19	10	9	19	9–30
	Complications	N	Death (PV occlusion and sepsis)	N	Open Necrosectomy and right hemicolectomy DVT arm from PICC	UTI from stent	Adhesions, cholangitis	N	—	—

All summary parameters are median (IQR). Severity of pancreatitis and definitions are defined by the Revised Atlanta Classification.^9^

*ERCP-induced pancreatitis with retroduodenal perforation.

†Required ERCP for cholangitis.

‡Seen as outpatient and electively admitted.

DVT indicates deep venous thrombosis; F, female; infected necrosis, infected necrosis was defined as positive tissue cultures or gas in the collection on computerized tomography imaging; IQR, interquartile range; M, male; MODS, multiorgan dysfunction syndrome; N, no; PICC, peripherally inserted central catheter; Y, yes.

The overall median (IQR) length of stay for this cohort was 98 (61–133) days, which included patient stay in the district hospitals prior to transfer. The median (IQR) time from hospital admission to PCD was 23 (19–34) days and time from PCD to SPRPN was 12 (2–30) days. The median (IQR) time of postoperative stay was 47 (19–102) days and time to collection resolution was 42 (36–49) days. The second patient died on a second admission from portal vein occlusion and sepsis related to other underlying disease. Three further patients had complications, namely peripherally inserted central catheter line deep venous thrombosis, urinary sepsis, and biliary sepsis requiring ERCP.

## DISCUSSION

We report the initial experience of 7 patients undergoing SPRPN for infected walled-off extra pancreatic necrosis. A SILS port has previously been described for direct access to pancreatic necrosis in 3 patients,^5^ its use into extrapancreatic collections is novel. Our patients were superselected, representing a serious clinical challenge but ultimately with a successful outcome using a 3-cm incision. Necrosis was cleared after only 1 procedure in all but the index case. Although the excess consumable costs are around $1040, overall savings might be seen in terms of reduced length of stay and surgical procedures.

Given the generally poor condition of these patients, it is unsurprising that 4 patients had complications. The second patient died from infected ascites following a portal vein thrombosis on a second admission. The fourth patient was seen to have infarcted their right colon from mesenteric artery inflammatory occlusion, seen once the necrosis was removed from the area, necessitating an open necrosectomy and extended right hemicolectomy.

The successful management of severe pancreatitis requires a personalized hybrid approach using a combination of available approaches. The technique described here should be considered an addition to the group of complex-minimal access procedures, which can be used in place of traditional open necrosectomy for difficult placed extrapancreatic WON.

## Acknowledgments

The authors thank NIHR Pancreatic Biomedical Research Unit Liverpool (Prof. R. Sutton, Department of General and Pancreatic Surgery, Liverpool University Hospitals NHS Foundation Trust, UK) for financial support of R.S. and Dr J. Evans (Department of Radiology, Liverpool University Hospitals NHS Foundation Trust, UK), who provided expertise and advice on interventional radiology.
